# Religious delusions in adolescence and young adults: Features of psychopathology and clinic

**DOI:** 10.1192/j.eurpsy.2021.2126

**Published:** 2021-08-13

**Authors:** V. Kaleda, U. Popovich, N. Romanenko

**Affiliations:** 1 Department Of Youth Psychiatry, FSBSI «Mental Health Research Centre», Moscow, Russian Federation; 2 Juvenile Psychiatry, Federal State Budgetary Research Center Mental Health Research Center, Moscow, Russian Federation; 3 Youth Psyhiatry, FEDERAL STATE BUDGETARY SCIENTIFIC INSTITUTION MENTAL HEALTH RESEARCH CENTER, Moscow, Russian Federation

**Keywords:** delusions, Religiosity, youth, psychosis

## Abstract

**Introduction:**

Religious delusions is a complex psychopathological phenomenon. The delusional disorders with religious content in young age, the need for an additional detailed study of the conditions of their formation, patterns of the course and outcome of the disease determine the relevance of this study.

**Objectives:**

To identify the psychopathological features, the conditions of formation, the characteristics of the course of psychosis with religious delusions in young age.

**Methods:**

95 patients (62 male and 33 female) with religious delusions (delusion of sin - 33,7%, delusion of demonic possession (40,0%), messianic and antagonistic delusion - 18,9%, oneiroid with religious content – 7,4 %) in psychotic episode (F20, F25 according to the ICD-10) at a young age (16-25 years) were included in the study and examined with clinical-psychopathological, clinical-follow-up and psychometric (PSP, SANS) methods. The average duration of follow-up was 7.4 ± 2.3 years.

**Results:**

In a post-psychotic period it is possible to preserve or form religiosity, as well as a complete reduction of the religious worldview in patients who had been indifferent to religious issues before the first episode of the disease. Though, the formation of residual psychotic symptoms with religious content were noted with greater frequency. The delusions of demon obsession in a psychosis episode is unfavorable prognostic factor.

**Conclusions:**

General psychopathological features of psychotic states with religious delusions, according to the specificity of young age, were identified. A role of the previous religiosity, including overvalued religious ideas, was clarified.

**Disclosure:**

No significant relationships.

